# Relative Values: Perspectives on a Neuroimaging Technology From Above and Within the Ethical Landscape

**DOI:** 10.1007/s11673-016-9725-1

**Published:** 2016-06-22

**Authors:** Gabrielle Samuel, Alan Cribb, John Owens, Clare Williams

**Affiliations:** 1 Brighton and Sussex Medical School, Falmer, East Sussex, UK; 2 Department of Educational Research, Lancaster University, Lancaster, UK; 3 Department of Education and Professional Studies, King’s College London, London, UK; 4 College of Business, Arts and Social Sciences, Brunel University London, Uxbridge, UK

**Keywords:** Empirical bioethics, fMRI, Vegetative state, Qualitative research, Ethics

## Abstract

In this paper we contribute to “sociology in bioethics” and help clarify the range of ways sociological work can contribute to ethics scholarship. We do this using a case study of an innovative neurotechnology, functional magnetic resonance imaging, and its use to attempt to diagnose and communicate with severely brain-injured patients. We compare empirical data from interviews with relatives of patients who have a severe brain injury with perspectives from mainstream bioethics scholars. We use the notion of an “ethical landscape” as an analogy for the different ethical positions subjects can take—whereby a person’s position relative to the landscape makes a difference to the way they experience and interact with it. We show that, in comparison to studying abstract ethics “from above” the ethical landscape, which involves universal generalizations and global judgements, studying ethics empirically “from the ground,” within the ethical landscape foregrounds a more plural and differentiated picture. We argue it is important not to treat empirical ethics as secondary to abstract ethics, to treat on-the-ground perspectives as useful only insofar as they can inform ethics from above. Rather, empirical perspectives can illuminate the plural vantage points in ethical judgments, highlight the “lived” nature of ethical reasoning, and point to all ethical vantage points as being significant. This is of epistemic importance to normative ethics, since researchers who pay attention to the various positions in and trajectories through the ethical landscape are unlikely to think about ethics in terms of abstract agency—as can happen with top-down ethics—or to elide agency with the agency of policymakers. Moreover, empirical perspectives may have transformative implications for people on the ground, especially where focus on the potential harms and benefits they face brings their experiences and interests to the forefront of ethical and policy discussion.

## Introduction

A growing body of literature highlights the limitations of abstract ethical reasoning for understanding the real-life ethical situations people face (Fox 1976; Haimes 2002; Samuel and Brosnan, 2011; Williams and Wainwright 2010). So called “empirical ethics” approaches, which deploy social science methods to investigate ethical issues, have emerged to account for this and have made a contribution to ethics scholarship (for example, see Fox 1976; Kleinman 1999; Haimes 2002). Moreover, by drawing attention to the potential harms and benefits faced by people in situ, such approaches may have transformative implications by bringing the experiences and interests of people on the ground to the forefront of ethical and policy discussion.

Within the broad current of empirical ethical work, some social scientists have more recently come to view themselves as undertaking “sociology in bioethics,” for example, by exploring the situated nature of ethical issues in clinical practice or in relation to innovative health technologies (for example, see Fox 1976; Williams et al. 2005). The construction sociology *in* bioethics is designed to evade the sub-disciplinary restrictions of either sociology *for* bioethics (an insider but essentially “servantile” conception of the role of sociology) or sociology *of* bioethics (an outsider and essentially critical conception). In this paper we aim to make a substantive contribution to sociology in bioethics and to help indicate and clarify the range of ways sociological work can contribute to ethics scholarship.

The substantive theme we have chosen to explore here concerns the use of an innovative neurotechnology, functional magnetic resonance imaging (fMRI^1^), in the attempt to diagnose and communicate with severely brain-injured patients. We draw upon empirical data from interviews with relatives of patients who have a severe brain injury, indicating how relatives construct and experience the ethical issues surrounding the use of fMRI technology and contrasting this with perspectives about the technology from mainstream bioethics scholars. We highlight the human-centred ethical perspectives found on the ground and ask about the potential benefits of empirically engaging with such perspectives.

In addition to defending the normative relevance of relatives’ perspectives (or other implicated parties), we wish to differentiate between approaches to empirical ethics which use “stakeholder” perspectives as a means of moving towards global ethical judgements (through theoretical or practical syntheses) and those which underline the plurality of ethical vantage points and question the traditional centre of gravity of ethical analysis. Whilst we see our findings as potentially relevant to both of these broad conceptions of empirical ethics, we are particularly eager to stress the distinctiveness and value of the latter conception.

This paper partly builds upon earlier work done by Cribb and colleagues (2008), which explored the idea of ethical “role positions” (Cribb et al. 2008). Based on interviews with translational research scientists and clinicians, the authors examined how the respective roles of these agents shaped their ethics. Since different professional and institutional settings have different cultural and social norms and expectations, the authors argued that the ethics of those within each setting needs to be understood as a product of the “ethical space” they occupy, that is, in terms of their “role position.” In this case, the scientists and clinicians constructed the relevant ethical issues differently and had different ethical obligation sets. The authors argued that this has important normative relevance:

Unless we understand the social construction of ethical positions … we will be unable to sensibly understand or attribute responsibility, or make judgements about what is defensible, or make informed recommendations about how things might be done better (359).

Here we wish to extend this reading to encompass all those who might be affected by, or implicated in, biomedical innovation. In particular, we want to argue that the role position of patients’ relatives is not only a valuable lens for understanding the ethics of fMRI use but also itself an important focus for ethics analysis.

In order to develop our points, we make use of an extended analogy: exploring ethics is like exploring a landscape, and a person’s position relative to the landscape makes a difference to the way they experience and interact with it. Specifically, we have in mind a rough contrast between two broad perspectives: exploration that is done through a global top-down process (akin to cartography) and exploration that is done from the position of a traveller situated within the landscape. The researcher in the analytic tradition of ethics tends to be engaged in a process which aims to create a general abstract map which objectively or neutrally represents the landscape from above; by contrast, social science researchers often examine or even adopt the position of the traveller situated within the landscape itself, paying attention to the socially embedded, context-specific and role-specific dimensions of ethics.

In this paper we outline the fMRI research and summarise the commentary that surrounds it within analytical bioethics. We then present our empirical methods and findings from the interviews with relatives, highlighting how they relate to, and diverge from, the more abstract ethical literature. In particular we consider one aspect of the mainstream ethical literature—the idea that society has a moral obligation to use fMRI for severely brain-injured patients given the potential that these patients may have some level of awareness (or “morally important competencies”). Some scholars have argued that identifying awareness levels of brain-injured individuals may have a direct impact on decision-making regarding treatment and prognosis (Bendsten 2013; Brukamp 2013). We compare this with the ways in which interviewees discuss the concerns they have with the technology. We suggest that the divergences between the literature and interviewees show the importance of taking role positions within the landscape seriously. We conclude by reflecting on the implications of this kind of research for the focus of, and approaches to, empirical ethics work.

## fMRI and Severe Brain Injury

Patients with a severe brain injury are generally classified as being in a vegetative state (VS) or a minimally conscious state (MCS). Clinicians define VS as “wakeful unresponsiveness.” Individuals in a VS have automatic functions such as being able to breathe on their own and having cycles of eye closure and opening (sleep-wake cycle), and may have reflexes such as a startle reflex or retracting from pain, but there is no sign of awareness and no evidence that the patient can perceive the environment or themselves, communicate with others, or form intentions. In a MCS, individuals fluctuate between levels of unawareness and levels of awareness of themselves and the environment—levels of awareness where they may have emotional responses to family members, say words or phrases and gesture, and/or show evidence of memory, attention and intention. However, awareness may be fleeting (Fins 2008). For both states, patients are usually independent of all machines apart from the one delivering artificial nutrition and hydration (which keeps VS and some MCS patients alive) (Report of a working party of the Royal College of Physicians 2003). VS and MCS may be transient stages in the recovery from coma or may persist until death. Prognosis is influenced by age, by the underlying cause, and by the state’s current duration. A little over half of those in a VS one month after a trauma will regain awareness, though with other causes, fewer than twenty per cent will recover after a month. The prognosis for MCS is more open than for a VS, but, for both states, the prognosis remains very limited for the majority of patients after being in VS or MCS for some time, even if they recover full consciousness.

The treatment of patients with severe brain injuries raises a range of social and ethical issues. Such issues often centre around clinical concerns such as patient management and the need for appropriate treatment and support. Concerns also relate to end-of-life decision-making. At present artificial nutrition and hydration can be withdrawn if an individual in the United Kingdom and the United States is deemed permanently vegetative. In the United Kingdom, the *Mental Capacity Act 2005* now applies to patients who are in an MCS. This Act states that decisions for individuals who lack capacity should be made based on the patient’s wishes, feelings, and values, even if they have not made a valid and applicable advance decision. At present there has only been one family who has brought this Act to the courts, and their application was rejected (Jackson 2013). There has been much debate surrounding this controversial decision both in the academic literature (Gillon 2012; Sheather 2013; Huxtable 2013; Johnston 2013; Mullock 2013) and in the media (Adams 2012). Moreover, the legal distinction between VS and MCS patients has been deemed ethically problematic (Kahane and Savulescu 2009). Johnson, for example, has argued that “consciousness should not preclude the withdrawal of life-sustaining treatment for minimally conscious patients any more than it does for other conscious patients” (Johnson 2010) because, for example, there is a possibility that life in a MCS—in which patients may be able to feel pain, have an emotional response and have insight into their plight—may be worse than in a VS (Demertzi et al. 2011). Fins and colleagues have also argued that there is no typical MCS or VS patient: each patient varies in their injury, diagnosis, and prognosis, and this is confounded by the fact that, firstly, diagnoses are not fixed but transient, and, second, misdiagnosis of the MCS is high (Fins 2006).

Diagnosis of the VS and MCS can be incredibly challenging and as many as forty per cent of individuals are diagnosed incorrectly (Andrews et al. 1996; more recently confirmed in Schnakers et al. 2009). In response, neuroscientists have recently made great efforts to try and assess brain function, mental state, and consciousness, using innovative neuroimaging technologies such as fMRI and positron emission tomography (PET)^2^ (Fernandez-Espejo et al. 2011; Goldfine et al. 2011; John et al. 2011). Such techniques are not therapies but rather aim to determine an individual’s level of retained brain activity or awareness by non-invasively providing images of the brain for analysis. Two particular pioneering studies have been widely reported. One 2006 study involved a twenty-three-year-old woman who had been diagnosed as vegetative (Owen et al. 2006). fMRI was used to scan the woman’s brain whilst she was asked to imagine different tasks. The researchers found that the patient’s neural responses were indistinguishable from those observed in healthy volunteers via fMRI. They concluded “this patient retained the ability to understand spoken commands and to respond to them through her brain activity” and “confirmed beyond any doubt that she was consciously aware of herself and her surroundings” (1402). In 2010, a subsequent paper by the same research group (Monti et al. 2010) reported on a similar study of fifty-four individuals. The authors reported that of the fifty-four,

… five were able to wilfully modulate their brain activity … In three of these patients, additional bedside testing revealed some sign of awareness, but in the other two patients no voluntary behaviour could be detected by means of clinical assessment. (579)

The researchers reported that one man, who at the time of scanning showed “signs of awareness … consistent with the minimally conscious state” (585), was able to use their technique to answer “yes” or “no” to questions during the fMRI. The authors concluded that “with further development this technique could be used by some patients to express their thoughts, control their environment, and increase their quality of life” (589).

These two studies, as well as other similar neurotechnological developments, sparked wide debate in the ethical literature about how such innovative technologies impact on severely brain injured individuals and their treatment. Below we explore this literature more closely and consider how the prominent concerns of scholars can be contrasted with interviewees’ views about the use of fMRI for severely brain-injured individuals.

## Commentary on the Use of fMRI Technology From Within the Analytic Bioethics Literature

The ethics of using fMRI technology for patients with severe brain injuries has been the subject of growing discussion within the top-down analytic bioethics literature: the use of such technology has been discussed with reference to the canon of well-established philosophical and legal principles. For instance, a prominent strand of discussion has centred around the nature of personhood. Scholars have questioned what it means to be a person and what it means to be conscious as well as questioning how we define such states, how we distinguish between the absence of consciousness and its minimal presence, and whether such states can be scientifically quantified through the use of neuroimaging techniques (for example, see Chien-Chang Wu 2008; Farah 2008; Schwartz and Schwartz 2008; Wilkinson and Savulescu 2008).

Another strand that has come to dominate the debate in the literature concerns how far fMRI challenges our existing beliefs about end-of-life decision-making for individuals with severe brain injuries. Many scholars have questioned the potential use of fMRI as a tool for asking those unable to overtly communicate whether they wish to live or die (Fisher and Appelbaum 2010; Sinnott-Armstrong 2011; Schwarzbauer and Schafer 2011). The literature includes a wide range of papers, from those which just raise the issue briefly to those which provide more detailed discussions (Bernat 2010; Fins 2010). Scholarship has been approached from a legal angle (Eisenberg 2008; Bressman and Reidler 2010; Fisher and Appelbaum 2010) and a philosophical perspective (Friedrich 2013). Some has provided a cautionary note. In the latter instance, scholars have warned that fMRI is still at an investigational stage with only preliminary results and that findings may not as yet be able to predict meaningful recovery (Wilkinson et al. 2009) or allow for communication about end-of-life decisions (Fins and Schiff 2010; Jox et al. 2012).

More recently, a number of papers in this literature have claimed that there is a moral imperative to develop and use fMRI technology for patients with severe brain injuries. For example, Bendsten (2013) argues that there:

… is an ethical obligation to use [fMRI, electroencephalograms^3^ or other similar technologies] to diagnose more accurately the patient diagnosed as having a disorder of consciousness, and that this obligation may stretch to investigate the possibilities for communication and potential decision-making capacity. (50)

Bendsten suggests that using fMRI technology may generate possibilities for communicating with patients in VS or MCS, creating the opportunity for receiving informed consent for further forms of treatment. As such, her argument is based on principles of respect for persons and respect for patient autonomy. On a similar basis, Brukamp has argued that in the developed world there is an ethical “obligation to perform fMRI on each patient with a chronic disorder of consciousness” (2013, 5) on the grounds that, where available, the technology is able to provide a more accurate diagnosis, which will be in the best interests of the patient.

Leaving aside the reasonableness and broad validity of these claims, these arguments provide good examples of the way in which top-down, global judgements are deployed in bioethics: they are generalizing judgements that appeal to abstract ethical principles and pertain to a non-specific and abstract ethical agent. For our current purposes, it is especially important to note the difference between invoking an abstract, hypothetical agent as opposed to invoking the actual agents who occupy the specific contexts and subject positions under consideration.^4^ From an abstract perspective it is possible to make broad in-principle judgements about singular ethical issues. From the position of real agents—however general and influential their role—practical judgements about traversing the ethical landscape need to be made, and these judgements occur in clusters rather than as singularities and are typically made in concert, negotiation, or conflict with other differently placed agents. Moreover, the practical ethical judgements made by real agents cannot be treated as neutral, objective, or impartial questions since these judgements must be considered within the substantive circumstances in which the agents concerned are immersed. It is to examples of real-life and situated ethical dilemmas that we now turn.

## Exploring Ethics on the Ground: Interviews With Relatives

In the following section we present empirical data taken from interviews with relatives of patients who have a severe brain injury. These data highlight some divergences between academic claims about the obligation to use fMRI in a clinical context and the concerns of families.

There is a very small population of individuals who not only have relatives with a severe brain injury but are also aware of the fMRI technology.^5^ Moreover, they are a particularly vulnerable population: they are hard to reach in terms of recruitment and are therefore under-represented in research studies. Family members with personal experience of this issue were reached via a brain injury support group. Six individuals expressed interest and were provided with an information sheet, and consent was gained to interview in each case (one interview was conducted as a joint session with husband and wife). As a qualitative study, it was not our intention to recruit a representative sample of participants for interview or to generalize the findings. Rather, the aim of qualitative study is to approach problems naturalistically, investigate the experience of specific, contextualized individuals, and discover the range of (often complex) views and beliefs individuals may have, by allowing participants to respond on their own terms and provide explanations (Carter et al. 2010). Qualitative techniques, whilst sacrificing statistical representativeness, can provide deeper and more original understanding of a complex issue (Carter et al. 2010) and, in this instance, of specific individuals within this often neglected population. Thus, whilst the experiences of our six participants are not generalizable, we will show how they serve to highlight new variables in the ethical debates about the use of fMRI for severely brain-injured individuals not discussed in the academic literature.

All six interviewees had considered fMRI for their own relative—in fact three of the participants’ relatives had undergone an fMRI. At the point of interview, three of the participants’ relatives remained in a vegetative or minimally conscious state, one had died while still in a disorder of consciousness, and one had regained full consciousness, although remaining severely disabled (see Table 1).

**Table 1:** Details of the sample (taken from Samuel and Kitzinger 2013)

Interviewee (pseudonym)	Patient (pseudonym)	Did the patient have fMRI	Results of fMRI (as reported by interviewee)	Time since injury (at time of interview)	Highest diagnosis reached (according to interviewee)
Alison	Andrew	Yes	No awareness detected	4 years	Permanent Vegetative State [PVS]
Eli	Ethan	Yes	Suggested some awareness	4 years	Minimally Conscious State (now deceased)
Trudy	Tracey	Yes	Suggested some awareness	2 years	Severely disabled
Rachel	Ronald	No – family wanted it, but patient not eligible	n/a	1.5yrs	PVS
Laura and Neil	Lavena	Suggested but not pursued by family	n/a	9 years	PVS

Interviews were semi-structured, face-to-face, and lasted between one and two hours; they were conducted by the first author. Interviews took place in a location of the participants’ own choosing (usually their home). It was important that we met away from the hospital setting and were not linked to the fMRI experimental studies in any way that might have inhibited what interviewees felt able to say. The interviews commenced with a broad discussion about participants’ experiences of having a relative with a disorder of consciousness and then went on to focus on participants’ understanding and opinions about the use of fMRI with such patients.

All interviews were audio recorded and fully transcribed. Transcriptions were thematically analysed using NVIVO (a qualitative data-analysis software package), with each transcript being systematically coded for issues such as how interviewees first heard about fMRI, their opinions about the pros and cons of fMRI, and the way they spoke about “hope.” In the interests of confidentiality, all names used in this article are pseudonyms. Due to the small population of individuals from which participants for this project were sourced, along with the particular vulnerability of these participants, some other identifying details have also been altered (for a review of the importance of this practice, please see Saunders, Kitzinger, and Kitzinger 2015).

Most obviously the perspective of families on the ground are not uniform but diverse. In addition, these perspectives are not wholly cognitive but are likely to be held and expressed with affect. For family members, these technologies represent a felt hope and risk, and the pros and cons surrounding them are not just held in intellectual balance but often experienced as emotional ambivalence. In the narrative below, we discuss the interviewees’ views about fMRI in a way which particularly highlights those participants’ beliefs least aligned to the debates raised in the academic literature. In this way we illustrate new variables so far not discussed in the ethical literature relating to this technology.

Some interviewees believed strongly in the use of fMRI for severely brain injured patients—“ideally you would think that patients in these conditions could undergo this scanning once a year, or something, to see if there were any changes” (Alison)—but this was not the case for all interviewees. For example, Rachel, who initially heard about the research in the news media when she was “looking for miracles,” still finds the technology “amazing” but is now much more sceptical of it. She did not want to pursue trying to access this technology for her husband, Ronald: “it’s really fascinating, it is fascinating, I would like to do it sometime but then there’s a lot of reasons—my reasons for not wanting to do it outweigh it at the moment”. These reasons seemed complex but crucially related to her concern that a negative finding from an fMRI scan would “prove against” Ronald and the continual struggle she had had for recognition that her husband was “in there.” Other interviewees, Laura and Neil, whose daughter had been in a vegetative state for nine years, had different reasons for not wanting to pursue fMRI:

It’s like a double edge sword because if we found that there was nothing there in a way that would’ve been easier. It’s kind of like a fifty-fifty chance: do we send Lavena there, does she have the tests done and then if she’s found to not react at all do we then think “right, yes, ok, diagnosis correct, Lavena is PVS.” But then or how do we handle it if there is something there but there’s not a damn thing that we can do to get to her to reach her…so it was very, very hard for us to weigh that up.

For this interviewee, her feelings of “dread” about the fMRI grew: “What if there was something going on in there? We’d feel so guilty that we haven’t tried harder to get through to her—but yet we know that everything has been done.” She concludes: “I don’t want to go there, I don’t want to put her or us through this—for different reasons—us emotionally and him physically”.

Relatives, of course, were not only concerned about their immediate families but had views with broader policy relevance. These broader concerns were grounded in their experiences but related more widely to the social resources, conditions, contexts, and consequences of technology use. Laura and Neil, for example, were worried about the more widespread use of fMRI: “I worry that other people may do it for their loved [ones]—their loved ones will have a scan—but then where do they go from there? What support is there for them?” Continuing, “you get a case of ‘yes, there might be something going on there,’ but actually ‘sorry but we can’t do anything about it.’”

These interviews with relatives demonstrate that making practical ethical judgements from within rather than above an ethical landscape is a complex business which may involve weighing a number of competing beliefs, emotions, pressures, and expectations. The responses given by participants demonstrate the important difference that positioning has for ethical decision making: whilst an analytically-based argument to pursue an innovative biomedical technology (such as those proposed by Bendsten and Brukamp and colleagues above) may on the surface seem straightforward and morally sound, such arguments can pay insufficient attention to the complex networks and levels through which technology is operationalized, the multiple agents and ethical positions involved, and thereby some of the key ethical, social, and emotional complexities of the whole ethical landscape.

Interviewees, for instance, highlighted the ways in which everyday practices and existing technologies can impact upon the care, comfort, and dignity of their relative. These reflections often emphasized the material constraints, experiential, and embodied effects and the practicability of technology usage. First, relatives of patients expressed reservations about the practicalities arising from the fMRI technology and the negative effect that a scan may have for the patient:

Even moving someone in Andrew’s condition, like the ambulance journey, etcetera, can be traumatic in a way that you or I couldn’t really understand. It’s very, very tiring and if there is any awareness there, it’s all change, it’s different, there are different people, different environment. Andrew doesn’t react terribly well to being put flat on his back—his limbs will stiffen and you can see that's almost a sign of protest. He does get used to it, but he can’t move, so he has to be moved from one bed to another, slid here, there and everywhere, it's all, it can be traumatic (Alison).

Do we want to put her through the upheaval of the movement when she has a routine and she’s comfortable? And then there was also the problem of the fact that she goes into spasm, and then she would need to be very completely still for the scans to work, and obviously they couldn’t sedate her because then that would be pointless, so there was so many different things that we had to think about (Laura).

Whilst concerns about access to the fMRI technology have been highlighted to some degree in the academic literature (for example, Tovino 2008), these interviewees’ narratives capture a much more personal, “lived” account of family beliefs and experiences of fMRI.

Second, relatives also expressed reservations about the technology on the grounds that an fMRI scan was unlikely to solve all, or even any, of the problems the families face, and given the practical realities of their situation, a scan may even create more problems beyond the initial test:

If you knew that your loved one could communicate … that would be great, then of course you would come to the situation of how do you accommodate that, the fact that you can do it in the scanner you can’t have someone in an MRI scanner for the rest of their life (Alison)

I think if you can say that somebody is responding then that is a very positive thing … But if you then ask them “Do you want to stay like this?” and you got the answer “no,” what would you do about it? … (Laugh) Nobody would ever dare ask that question. Because that is the big issue isn’t it? And that’s where euthanasia is … (Eli)

Similarly to Eli, academics have widely questioned the potential effect of fMRI during end-of-life decision-making and on decision-making in general. Academics and scientists are also acutely aware of the drawback of fMRI as an immoveable instrument—in fact, neuroscientists have begun exploring the use of electroencephalography (EEG) as an alternative (Goldfine et al. 2011; John et al. 2011). However, the academic and family discourses diverge in their consideration of whether or not to pursue this technology, with scholars focusing much less of their attention on how the limited capabilities of the fMRI technology are experienced by and emotionally impact on families. This point is nicely opened up in one of Rachel’s comments:

It’s not like something we can carry around with him…what if you do get in there and then you’ve got to take him away again … I find that really hard. I thought, “Well, what if you do get in there and then you’ve got to take him away again? What if you get that little glimpse of him?” It’s not like you can live in the fMRI … and then it’s gone … No I don’t want one. (Rachel)

Narratives such as this one and those above reveal that relatives not only view the use of fMRI as unable to solve the existing issues facing patients but also as very likely to create new ethical problems. They highlight the importance of considering how the drawbacks of fMRI can impact on families before obligating such families to consent to the use of the technology.

Finally, separate to their concerns about the fMRI scanning, some of the interviewees spoke at some length about their experiences in hospitals and the need for basic care, support, and access to facilities. Whilst these concerns did not detract from their views about research and the possibilities that fMRI could hold, it did seem at times that maintaining an adequate standard of dignity and care for patients weighed more heavily on their lives, and in a sense was either equally, or more important, to them than this new technology:

I can remember coming in [the hospital] one day. She’s on the ward and—no kidding—bearing in mind Tracey can’t move herself, her arm was through the thing like this [shows arm], and her head was scrunched between the metal bar, scrunched there and just laying there, and I went ballistic, you know I said “there’s a nurse sitting over there, can’t she see she’s stuck” It was dreadful—she’d always come back with bed sores (Trudy).

Dignity is everything isn’t it, if you lose your dignity it’s very difficult, and for me to see Andrew without any dignity, it’s very hard, people need to be aware of that (Alison).

Interviewees reported breakdowns in care for their relative due to a lack of staff expertise with existing equipment (for example the hoists used to move patients around) and failures to provide or repair it (for example, obtaining and maintain appropriate wheelchairs). They were acutely aware of the context of care delivery and thus sometimes questioned what fMRI could deliver in practice, even if the scientists were successful in their ambitions to develop its capability in ideal laboratory settings. Indeed, this finding reflects other research with relatives of patients with a severe brain injury where the recruitment method was not related to fMRI (Kitzinger and Kitzinger 2012).

## Discussion

We have noted some of the differences between the perspectives of individuals who have a severely brain-injured relative and the perspectives found in the more abstract and generalized academic literature. Of course we could have rehearsed perspectives from multiple other real world agents—researchers, clinicians, budget holders, journalists, policymakers, and so on. Each of these sets of perspectives would illuminate both specific aspects of and specific angles on the large and densely organised ethical landscapes in which the relevant ethical issues are embedded. Different positions within and trajectories across the landscape will produce different kinds of accounts. What seems salient will vary, as will the nature and magnitude of costs and benefits of the technology and the texture of the ethical issues it raises.

These perspectives from the ground can contribute to understanding normative ethics in a range of ways. Here we will briefly contrast four such ways, each indicating partly complementary and partly competing emphases in empirical ethics. First, they can be treated as feeding into and moderating “ethics from above” by, for example, being used in reflective equilibrium approaches to empirical ethics. That is, perspectives from the ground provide evidence of, and formulations of, first-order intuitions in reflective equilibrium approaches to empirical ethics (Rawls 1971; van Thiel and van Delden 2010). This move partially grounds and thereby refines “global” ethical readings and synthesizes diverse perspectives together. Second, there are analogous approaches to empirical ethics that seek to achieve a more practical kind of synthesis between perspectives by bringing diverse parties together in deliberative democratic exercises or smaller scale processes of mutual deliberation and negotiation, such as is found in “dialogical ethics” approaches (for example Kim et al. 2009; Widdershoven, Abma, and Molewijk 2009). It is clear that attention and responsiveness to the relatives’ perspectives outlined above could play an important role in such theoretical or practical syntheses. But empirical ethics approaches can also be used—third—not for synthesising but for positively emphasizing and exploring the diversity of experiences and perspectives in ethics and—fourth—for questioning the traditional centre of gravity of ethical scholarship. It is the latter two emphases that we wish to say a little more about here.

An understanding of ethics can only be partly accomplished by synthesising aspirations; it also depends upon being ready to co-travel with multiple agents and experience the features, contours, and challenges of the landscapes from their perspectives. It needs to be stressed that this is not simply a question of appearances—how things look to various agents—nor are these perspectives and concerns simply relevant “data.” Different things matter in different instances, and there is not simply one over-arching ethical question but innumerable ethical issues divided across differently placed agents. For example, family members have ethical decisions to make about how much to initiate or cooperate with attempts to use fMRI imaging in the case of their brain-injured relatives. For them there is comparatively little to be gained by appealing to general arguments for some over-arching ethical imperative. They will rightly be concerned about the specific circumstances and cultures of care their family members are, and might be, encountering. They will also often be—in common with clinicians—directly alive to questions about the material constraints of technologies and to questions about practical feasibility and consequences. This indicates an additional substantive reason for engaging in empirical ethics: by drawing attention to the diverse variety of issues that matter to agents situated within ethical landscapes, empirical ethics provides a way of recognizing, respecting, and representing people on the ground. Where empirical ethics is able to bring the experiences, interests, and views of people on the ground to the forefront of ethical and policy discussion—and thereby to complement, modify, or even displace official or dominant perspectives—it has the potential to be transformative for these people. In addition, by recentring ethics analysis around the ethical burdens and dilemmas that have to be navigated on the ground, it offers normative as well as explanatory insights.

Academic arguments for a general imperative to deploy fMRI are, of course, still valuable, especially if they are qualified and inflected by attention to the perspectives of travellers through the relevant landscape. These general arguments certainly have relevance to the ethical judgements made by policymakers (the real world near-analogue of the abstract agents associated with the top-down or synthesizing perspective). However, if such policymakers want to make defensible ethical decisions, they need to make them whilst also being responsive to and ideally in conversation with other actual agents. Attention to the various positions in, and trajectories through, the ethical landscape makes ethics researchers much less likely to equate the abstract agency of top-down ethics with the agency of policymakers. For a technology to be operationalized requires a complex network of differently located agents of which policymakers are just one part. None of these sets of agents, including policymakers, are simply making one big in-principle ethical judgement about the defensibility and desirability of technology usage. Rather, as dialogical approaches to ethics recognize, each set of agents is in relationships with, and has ethical obligations to, other agents. These obligations include treating the ethical concerns of other agents with significance—not only because they help to illuminate one portion of the full ethical landscape but also on their own terms as of substantive ethical importance.

In summary, “sociology in bioethics” can inform ethics from above, but, as we are stressing here, it can also illuminate the plural forms of, and vantage points in, ethics—the inherent diversity, complexity, and situatedness of ethical experiences, dilemmas, and judgements. Sociological contributions can also serve to question the framing of ethics scholarship, including the presumed centrality of certain agendas; for instance, why the dilemmas of abstract global agents or relatively powerful policymakers and professionals are frequently located at the centre of the discipline and the dilemmas of relatives, for example, are typically treated as marginal.^6^

## Conclusion

Advances in biomedical technology, including neurotechnology, notoriously give rise to new ethical challenges. Some of these ethical challenges arrive at the doorstep of members of the public who just happen to be in the right (or wrong) place at the right time. Ethics scholarship has much to gain by devoting proper attention to the way ethical issues and dilemmas are experienced and negotiated by ordinary persons such as the relatives considered in this research. Such attention could have transformative implications for patients and families by placing their experiences, interests, and views at the forefront of ethical and policy discussion. For these reasons, we want to suggest, it is important not to treat the study of on-the-ground perspectives such as those of the relatives investigated here as of secondary importance and as simply “informing” serious abstract ethics from below.

Rather, empirical ethics has the potential to confront important substantive ethical questions that might otherwise be neglected and to illuminate the nature of ethics *as lived*. Studying ethics from above the ethical landscape—aspiring towards universal generalizations and global judgements—often has the character of rarefied abstract reasoning. Studying ethics within the ethical landscape foregrounds a more plural and differentiated picture: it reveals a closer and more detailed account of interlocking networks of multiple social conditions and agents and brings with it various kinds of “weight”—related, for example, to practicability, materiality, and embodied affect. This kind of focus is of epistemic importance to normative ethics. If we seriously want to consider the ethics of neuroimaging technology, for example, we need to be ready to adopt the role positions of the relatives considered here and ask ourselves what we think we ought to do in the various situations in which they find themselves. This does not make it invalid to ask the more general, in principle, question about whether there is an obligation for particular health systems to make such technology available. But it does mean we are also much more likely to be able to answer this latter question with greater wisdom.

## Footnotes

1 fMRI is a brain scanning technique which measures brain activity by detecting associated changes in blood flow to different areas of the brain

2 PET is an imaging technology that produces three-dimensional images of the functional processes within the body. It does this by imaging the body after a biologically active (tracer) molecule is introduced to the body

3 Otherwise known as EEG, this is a test that can detect abnormalities related to electrical activity of the brain.

4 The ethical position of some actual agents—perhaps specific policy makers or senior professionals in relatively powerful roles—may approximate to that of an abstracted agent but this similarity is deceptive.

5 It was particularly important that the interviewees were aware of the fMRI technology prior to the interview. This ensured that false hope was not given to any of the interviewees, some of whom were vulnerable, during the interview process.

6 For example, see the feminist or “naturalized” bioethics literature such as Lindeman, Verkerk, and Urban Walker 2008
